# EMR-Documented Contraception for Patients Prescribed Medications With Adverse Perinatal Outcomes

**DOI:** 10.1001/jamanetworkopen.2024.23930

**Published:** 2024-07-22

**Authors:** Joseph V. Nolan, Marisa A. Muhonen, Michael A. Jaeb, Michael G. Semanik, Wendy N. Nembhard, Zachary N. Stowe

**Affiliations:** 1 Department of Psychiatry, University of Wisconsin–Madison; 2 School of Medicine and Public Health, University of Wisconsin–Madison; 3 Department of Epidemiology, Fay W. Boozman College of Public Health, University of Arkansas for Medical Sciences, Little Rock

## Abstract

This cross-sectional study examines the rates of method of contraception documentation in the electronic medical record (EMR) for patients receiving 1 of 3 drugs known to be associated with adverse perinatal outcomes.

## Introduction

Unintended pregnancy is a national health concern accounting for over 45% of pregnancies in the United States.^1^ Many women in their reproductive years may receive treatment with medications associated with adverse perinatal outcomes in the absence of method of contraception documentation (MCD) in the electronic medical record (EMR).^2^ Such medication exposures occur during organogenesis, and treatment options and/or interventions are subject to availability that is influenced by each state’s political landscape.^3^ Modifications to the EMR could reduce risk and enhance clinician engagement. Previous research has shown that rates of MCD and counseling for women taking teratogenic medications increase significantly after educating clinicians about how to properly document this information in the EMR.^4^ Hard stops in the EMR have proved to be an effective tool in improving patient process outcomes.^5^ This study examined EMR-documented MCD for women seen in outpatient clinics, focusing on 2 known teratogens (lithium and valproate) and a widely prescribed medication associated with adverse perinatal outcomes (gabapentin).

## Methods

The University of Wisconsin–Madison Minimal Risk Research institutional review board classified this study as exempt because secondary research was used; no consent was required. This cross-sectional study followed the STROBE reporting guideline. Using SlicerDicer within EpicCare Ambulatory, we examined the MCD for individuals who self-identified as female in the EMR, were between 12 and 50 years of age, and were seen by a University of Wisconsin Health clinician in an outpatient clinic between August 1, 2019, and August 1, 2022. SlicerDicer queries access anonymous patient populations, providing numeric results based on selected parameters. This cohort was divided into 3 different age groups: teenagers (12-17 years), adults (18-34 years), and women of advanced maternal age (35-50 years). No race or ethnicity data were used. An MCD included contraception as defined by EpicCare and procedures associated with sterilization. Abstinence, family planning, and sexual orientation were not listed as means of contraception in EpicCare. Medication groups were defined as patients receiving a prescription for any form of lithium, valproate, or gabapentin or none of these medications. The χ^2^ and relative risk analyses were performed February 5 to February 28, 2024, using IBM SPSS Statistics, version 29, with a 2-sided *P* < .05 for statistical significance.

## Results

A total of 511 547 individual outpatient records of patients aged 12 to 50 years who self-identified as female were included. Only 24.4% had an MCD in the EMR. The proportions of women with an MCD taking lithium, valproate, or gabapentin or none of these medications are given in the Table. For example, women aged 18 to 34 years (32.4%) compared with women aged 12 to 17 years (3.9%) (χ^2^_1_ = 15483.26; *P* < .001) and women aged 35 to 50 (22.3%) (χ^2^_1_ = 7373.79; *P* < .001) were significantly more likely to have an MCD (vs not). Also, women taking lithium, valproate, or gabapentin (39.1%) compared with women not taking these medications (23.8%) were significantly more likely to have an MCD (vs not) (χ^2^_1_ = 2460.79; *P* < .001). Women taking lithium (41.6%) compared with women taking valproate (30.6%) were significantly more likely to have an MCD (vs not), (χ^2^_1_ = 36.61, *P* < .001), and women taking gabapentin (39.7%) compared with women taking valproate (30.6%) were significantly more likely to have an MCD (vs not) (χ^2^_1_ = 46.36, *P* < .001).

**Table.** Proportion of Women of Childbearing Years With an MCD

Age group, y	Women taking lithium			Women taking valproate			Women taking gabapentin			Women not taking medication	
	Total No. (%)	With MCD, No. (%)	RR (95% CI)^a^	Total No. (%)	With MCD, No. (%)	RR (95% CI)	Total No. (%)	With MCD, No. (%)	RR (95% CI)	Total No. (%)	With MCD, No. (%)
12-17	27 (2.0)	7 (25.9)	6.86 (3.62-12.99)	120 (8.3)	12 (10.0)	2.64 (1.54-4.53)	319 (1.8)	68 (21.3)	5.64 (4.54-7.00)	45 641 (9.3)	1726 (3.8)
18-34^b^	537 (40.5)	292 (54.4)	1.72 (1.59-1.86)	548 (37.9)	230 (41.0)	1.33 (1.20-1.46)	5404 (31.2)	3121 (57.8)	1.82 (1.78-1.87)	208 407 (42.4)	66 003 (31.7)
35-50	763 (57.5)	253 (33.2)	1.60 (1.45-1.77)	778 (53.8)	200 (25.7)	1.24 (1.10-1.40)	11 573 (66.9)	3670 (31.7)	1.53 (1.49-1.57)	237 430 (48.3)	49 187 (20.7)
Overall	1327 (0.3)	552 (41.6)^c^	1.75 (1.64-1.86)	1446 (0.3)	442 (30.6)	1.29 (1.19-1.39)	17 296 (3.4)	6859 (39.7)^d^	1.67 (1.64-1.70)	491 478 (96.1)	116 916 (23.8)^e^

Abbreviations: MCD, method of contraception documented; RR, relative risk.

^a^ Women not taking any of the 3 medications were the reference group for the RR analyses.

^b^ Women aged 18 to 34 years (32.4%) compared with women aged 12 to 17 years (3.9%) (χ^2^_1_ = 15483.26; *P* < .001) and women aged 35 to 50 (22.3%) (χ^2^_1_ = 7373.79; *P* < .001) were significantly more likely to have an MCD (vs not).

^c^ Women taking lithium (41.6%) compared with women taking valproate (30.6%) were significantly more likely to have an MCD (vs not) (χ^2^_1_ = 36.61; *P* < .001).

^d^ Women taking gabapentin (39.7%) compared with women taking valproate (30.6%) were significantly more likely to have an MCD (vs not) (χ^2^_1_ = 46.36; *P* < .001).

^e^ Women taking lithium, valproate, or gabapentin (39.1%) compared with women not taking lithium, valproate, or gabapentin (23.8%) were significantly more likely to have an MCD (vs not) (χ^2^_1_ = 2460.79; *P* < .001).

## Discussion

Despite the well-documented risks,^6^ women are prescribed valproate even though they are significantly less likely to have an MCD compared with women prescribed either lithium or gabapentin. It is reassuring that women prescribed any of these medications were significantly more likely to have an MCD compared with the group not prescribed a medication; this finding was consistent across all age groups. A limitation is that SlicerDicer does not have access to prescriptions obtained outside the University of Wisconsin Health system unless patients report those prescriptions. The reproductive safety for medications may not be well delineated when the medication is first approved for use and may change with postmarketing surveillance. Furthermore, treatment planning may be influenced by the fluidity of the political impact on health care.^3^ Strategies to mitigate the risk of potential teratogenic exposures could involve harmonization across EMR versions or health care systems, including linkage of EMR alerts and hard stops to use up-to-date safety data to enhance clinician education and potentially reduce risk. Although such measures add to clinician burden, safeguarding fetal development is invaluable. Identifying opportunities where the EMR is uniquely equipped to enhance clinician education and improve outcomes warrants attention.

## Notes

Supplement.
Data Sharing Statement
