# Supplementary material for: EMR-Documented Contraception for Patients Prescribed Medications With Adverse Perinatal Outcomes
Source: JAMA Netw Open. 2024 Jul 22;7(7):e2423930. doi: 10.1001/jamanetworkopen.2024.23930 (PMC11265134; doi:10.1001/jamanetworkopen.2024.23930)
Supplement: Supplement. — Data Sharing Statement [file jamanetwopen-e2423930-s001.pdf]

Data Sharing Statement

Nolan. EMR-Documented Contraception for Patients Prescribed Medications With Adverse Perinatal Outcomes. *JAMA Netw Open*. Published July 22, 2024.
doi:10.1001/jamanetworkopen.2024.23930

Data
Data available: No

Additional Information
Explanation for why data not available: Data will not be shared at this time for the interest of time efficiency as well as potential limitations on the proprietary use of any software products (i.e., Epic licensing of SlicerDicer). All relevant frequency data are located within the manuscript.
